# Evidence of hidden diversity and taxonomic conflicts in five stream fishes from the Eastern Zimbabwe Highlands freshwater ecoregion

**DOI:** 10.3897/zookeys.768.21944

**Published:** 2018-06-19

**Authors:** Albert Chakona, Wilbert T. Kadye, Taurai Bere, Daniel N. Mazungula, Emmanuel Vreven

**Affiliations:** 1 South African Institute for Aquatic Biodiversity, Private Bag 1015, Grahamstown, South Africa, 6140; 2 Department of Ichthyology and Fisheries Science, Rhodes University, P.O. Box 94, Grahamstown, South Africa, 6140; 3 School of Wildlife, Ecology and Conservation, Chinhoyi University of Technology, P. Bag 7724, Chinhoyi, Zimbabwe; 4 Royal Museum for Central Africa, Section of Vertebrates, Ichthyology, Leuvensesteenweg 13, 3080, Tervuren, Belgium; 5 KU Leuven, Department of Biology, Laboratory of Biodiversity and Evolutionary Genomics, Deberiotstraat 32, 3000 Leuven, Belgium

**Keywords:** *Amphilius*, candidate species, *Chiloglanis*, cryptic diversity, DNA barcoding, fish, freshwater, GMYC, *Hippopotamyrus*, *Zaireichthys*

## Abstract

Stream fishes of the Eastern Afromontane region are among the least studied vertebrates in this region, despite the potential for harbouring cryptic diversity. The present study examined mitochondrial cytochrome oxidase subunit I (COI) sequence divergence in 153 specimens of stream fishes belonging to four genera and three families, [(*Amphilius* and *Zaireichthys* (Amphiliidae); *Chiloglanis* (Mochokidae); and *Hippopotamyrus* (Mormyridae)], in the Eastern Zimbabwe Highlands (EZH) freshwater ecoregion to explore the extent to which the current taxonomy conceals the ichthyofaunal diversity in the region. The General Mixed Yule Coalescent (GMYC) species delineation method identified 14 clusters within five currently recognised ‘species’ from the EZH ecoregion. Only one of these clusters represents a named species, while 13 of them represent candidate or undescribed species. Our results revealed that effective conservation of this region’s unique biota is limited by the incomplete knowledge of taxonomic diversity and inaccurate mapping of species distribution ranges.

## Introduction

The Eastern Zimbabwe Highlands (EZH) freshwater ecoregion (Thieme et al. 2005; Abell et al. 2008), also referred to as the Manica Highlands (Clark et al. 2017), is renowned as a centre of floral diversity and endemism, harbouring approximately 150 endemic plant species (Clark et al. 2017; Van Wyk and Smith 2001). However, to date, the EHZ’s ichthyofaunal diversity is considered comparatively low. Marshall (2011) listed 25 freshwater fishes for the EZH freshwater ecoregion, of which one species, *Labeobarbus pungweensis* (Jubb, 1959), is endemic to this ecoregion. The continued recognition of many of the stream fishes from the EZH freshwater ecoregion as having wide geographic ranges (see Skelton 2001; Marshall 2011) demonstrates the poor taxonomic attention and lack of systematic knowledge in this region and the broader southern African subregion. Growing evidence from previous and ongoing DNA-based studies shows that many species of freshwater fishes that were previously considered to have wide geographic ranges are instead complexes comprising several genetically divergent lineages (e.g., Chakona et al. 2013; Goodier et al. 2011; Kramer et al. 2004; Kramer and Wink 2013; Linder et al. 2010; Swartz et al. 2007, 2009). These studies have stimulated renewed interest in the systematics of freshwater fishes in the region as evidenced by the recent revalidation of some junior synonyms as well as the identification and description of several new species (e.g., Chakona and Swartz 2013; Chakona et al. 2014; Chakona and Skelton 2017; Kramer et al. 2003, 2007, 2012, 2013, 2014; Kramer and Swartz 2010; Kramer and van der Bank 2011; Maake et al. 2014). There is thus need for expanding the application of molecular approaches as these may lead to the identification of presently unrecognised diversity and provide insights on the patterns of endemism of stream fishes from other understudied regions in southern Africa such as the EZH freshwater ecoregion.

**Figure 1.**
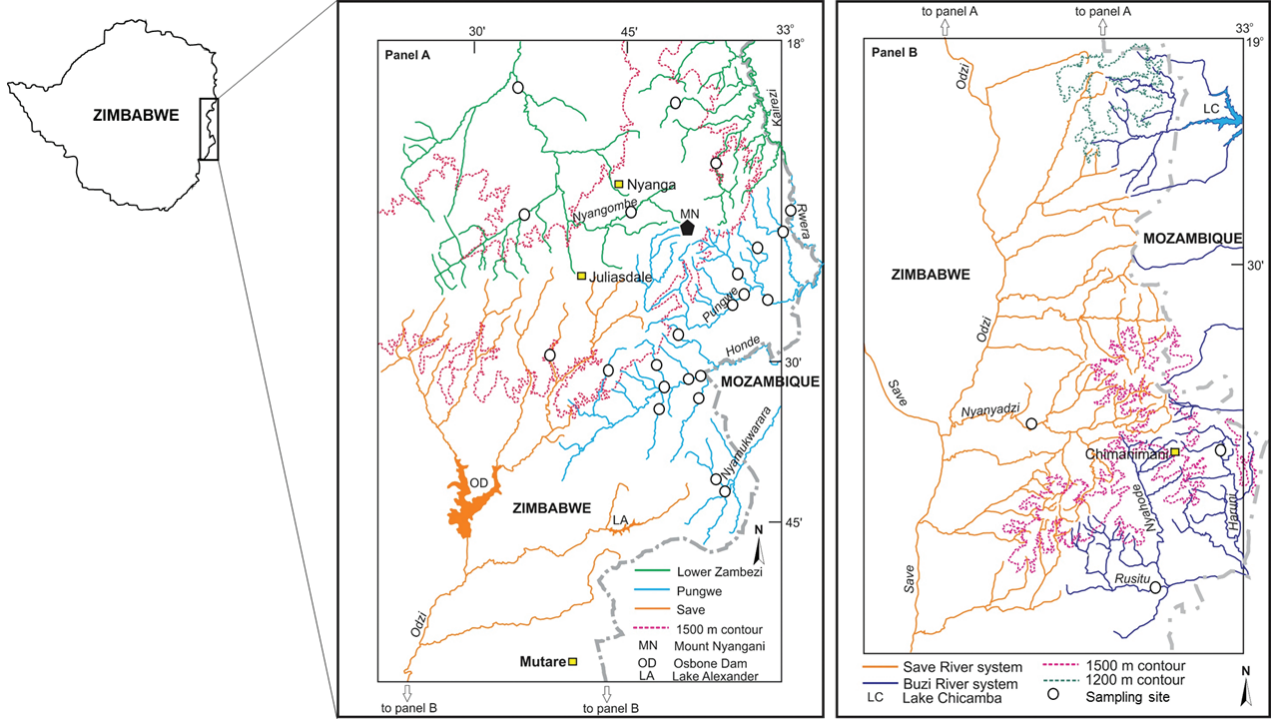
Map of the Eastern Zimbabwe Highlands (EZH) freshwater ecoregion showing the river systems (Lower Zambezi, Pungwe, Buzi and Save) that drain this region and the sampling localities for the present study.

To examine the hypothesis that the perceived broad geographical ranges for many stream fishes in the EZH freshwater ecoregion may be due to overlooked diversity, the present study used genetic data to explore the possible existence of species level differentiation in fishes from tributaries of four river systems, the Lower Zambezi, Pungwe, Save and Buzi, in this ecoregion (Fig. 1). The present study used the mitochondrial cytochrome oxidase subunit I (COI) gene to examine the patterns of intraspecific divergence within five morphologically defined fish species, *Chiloglanis neumanni* Boulenger, 1911, *Amphilius natalensis* Boulenger, 1917, *A. uranoscopus* (Pfeffer, 1889), *Zaireichthys monomotapa* Eccles et al., 2011 and *Hippopotamyrus ansorgii* (Boulenger, 1905), from sub-catchments of the lower Zambezi River system (the Nyangombe and Kairezi rivers and their tributaries), the Pungwe River system and its tributaries (the Rwera, Pungwe mainstream, Honde and Nyamukwarara rivers), tributaries of the Buzi River system (the Haruni and Rusitu rivers) and the Save River system and its tributaries (the Odzi and Nyanyadzi rivers) (Fig. 1). These taxa were selected due to uncertainties about their taxonomic status (Marshall 2011), their perceived broad geographic distribution ranges that are surprising considering recent evidence from studies of other freshwater fishes that were previously thought to be wide ranging species (e.g., Chakona and Skelton 2017; Decru et al. 2012, 2013, 2015; Zengeya et al. 2011), as well as their peculiar disjunct distribution patterns (i.e., discontinuous and separated by wide geographic distance) in the case of *A. natalensis* and *H. ansorgii* (Marshall 2011; Skelton 2001).

The taxonomic uncertainty of the suckermouth catlet of the EZH freshwater ecoregion has persisted for decades. The first published detailed checklist of freshwater fishes of Zimbabwe by Jubb (1961) contained one species of suckermouth catlet, identified as *Chiloglanis neumanni*, despite the enormous geographic distance between the EZH ecoregion and the type locality of *C. neumanni* which is in the Upper Bubu River (Rufiji River system) in Tanzania (Boulenger 1911; Eschmeyer et al. 2018). Bell-Cross and Minshull (1988) listed two species of *Chiloglanis* from the EZH ecoregion that were identified as *C. emarginatus* Jubb & Le Roux, 1969 and *C. neumanni*. Distribution maps in Skelton’s (2001) guide to the freshwater fishes of southern Africa suggested that there are three species of suckermouth catlets in the EZH ecoregion, *C. emarginatus*, *C. neumanni* and *C. pretoriae* van der Horst, 1931. *Chiloglanis emarginatus* was described from the Lekkerloop River in the Inkomati River system in South Africa (Jubb and Le Roux 1969). The distribution of this species was previously divided into northern (Pungwe and Lower Zambezi Rivers) and southern (Inkomati and Phongolo Rivers) populations (Skelton 2001). Engelbrecht et al. (2007) subsequently considered records of the northern population to have been incorrectly identified, and indicated that *C. emarginatus* was restricted to the Inkomati and Phongolo River systems. *Chiloglanis pretoriae* was described from the Crocodile River, a tributary of the Limpopo River system in South Africa. More recently, Marshall (2011) summarised the existing knowledge of the fishes of Zimbabwe and indicated that *C. pretoriae* does not occur in the EZH ecoregion as the Limpopo River system is the northern-most distribution limit for this species. Marshall (2011) also commented that *C. neumanni* is unlikely to occur in Zimbabwe, and highlighted the need for detailed evaluations to determine the diversity and taxonomic integrity of suckermouth catlets in the country.

Similar to *Chiloglanis*, the species of *Amphilius* in the EZH freshwater ecoregion also continue to be surrounded by taxonomic uncertainty. Jubb (1961) listed a single species of *Amphilius* for this ecoregion, and identified it as *A. grandis* Boulenger, 1905, originally described from the Tana River system in Kenya. In their review of the Amphiliidae of southern Africa, Bell-Cross and Jubb (1973) recognised three species of *Amphilius* from the EZH ecoregion: *A. platychir* (Günther, 1864), originally described from Sierra Leone, west Africa; *A. lampei* Pietschmann, 1913, originally described from the Ethiopian Highlands near Harar, east Africa, and *A. natalensis* Boulenger, 1917, originally described from the Kranzkloof River, which is part of the Umgeni River system in South Africa. In a comprehensive revision of *Amphilius* species from west, east and southern Africa, Skelton (1984) considered *A. grandis* to be a junior synonym of *A. uranoscopus* (Pfeffer, 1889), a species which was originally described from the Wami River system in Tanzania. In this revision, Skelton (1984) presented unequivocal evidence that supported Günther’s (1902) assertion that *A. platychir* was restricted to west Africa, and transferred the southern African specimens that were previously identified as *A. platychir* to *A. uranoscopus*. Skelton (1984) further considered that Bell-Cross and Jubb’s (1973) identification of mountain catfish specimens from the Nyazengu River in the Eastern Highlands as *A. lampei* was erroneous, and transferred these specimens to *A. natalensis*. These taxonomic changes were followed by subsequent authors (Bell-Cross and Minshull 1988; Marshall 2011; Skelton and Teugels 1986; Skelton 2001). Skelton (1984) also described a new species, *A. laticaudatus* from the Lower Buzi in Mozambique. This species is currently only known from its type locality. Thus, *A. uranoscopus* and *A. natalensis* are the only two mountain catfish species that are currently recognised from the EZH freshwater ecoregion (Marshall 2011; Skelton 1984, 2001). However, a recent taxonomic revision of *A. uranoscopus* complex in Kenya and Tanzania by Thomson and Page (2010) resulted in the resurrection of two species, *A. grandis* and *A. krefftii*, which were both previously considered to be junior synonyms of *A. uranoscopus*, as well as the description of a new species, *A. athiensis* Thomson & Page (2010). Occurrence of three distinct species in such a small geographic area, which represents only a very small portion of the *A. uranoscopus* sensu lato distribution, raises the possibility that this complex may contain many undescribed species across its current wide geographic range. It is also likely that *A. uranoscopus* sensu stricto may not even occur in southern Africa. If this is ascertained, investigations will need to be made to determine the taxonomic status of the three synonyms of *A. uranoscopus* from southern Africa, *A. hargeri* Boulenger, 1907, *A. brevidorsalis* Pellegrin, 1919 and *A. cubangoensis* Pellegrin, 1936 and identify possible new species.

The genus *Zaireichthys* contains the smallest catfishes in Africa, with a total of 18 species (Eschmeyer et al. 2018). The EZH freshwater ecoregion is currently thought to harbour a single valid species of sand catlets, *Z. monomotapa*, originally described from the Save River system (Eccles et al. 2011), the largest river basin in Zimbabwe. The species is currently considered to be widely distributed in tributaries of the middle and lower Zambezi, Pungwe, Buzi and Save River systems in Zimbabwe (Marshall 2011). However, the discovery of substantial levels of intraspecific genetic differentiation within almost all wide-ranging stream fishes studied thus far in southern Africa raises the possibility that *Z. monomotapa* may also contain hidden diversity.

There are uncertainties about the origin of the specimens that were used for the original description of *Hippopotamyrus ansorgii* as the type locality is vaguely described as ‘between Benguella and Bie’ (Boulenger 1909). This region encompasses the Angolan highlands which are drained by at least five river systems, with the Kwanza, Upper Zambezi, Okavango and Kunene being the four major systems. Kramer and Swartz (2010) postulated that the Kunene could probably be the type river for *H. ansorgii*. For a long time, *H. ansorgii* was the only recognised species of the genus *Hippopotamyrus* in southern Africa, with a disjunct distribution broadly divided into western (Kwanza, Kunene, Okavango and Upper Zambezi) and eastern (lower Zambezi, Pungwe and Buzi) populations (Skelton 2001). Recently, integrated systematic studies uncovered the existence of deeply divergent lineages within *H. ansorgii*, which led to the description of two new species, *H. szaboi* from the Upper Zambezi (Kramer et al. 2004) and *H. longilateralis* from the Kunene River system (Kramer and Swartz 2010). These findings, coupled with the wide geographic gap between the western (i.e., the type region) and eastern populations of *H. ansorgii*, raise the possibility that the populations of the EZH freshwater ecoregion may be genetically distinct, and potentially represent previously unrecognised species.

In the present study, extensive sampling of *C. neumanni*, *A. natalensis*, *A. uranoscopus*, *Z. monomotapa* and *H. ansorgii* was done from 27 localities in the EZH freshwater ecoregion (Fig. 1), and mitochondrial COI sequences were generated to determine the degree of genetic distinctiveness of these populations and identify unique lineages. The study included additional sequences downloaded from BOLD and, where available, topotypes (conspecific specimens collected from or in the vicinity of the type locality) were included in the analyses for comparisons. This study represents the first comprehensive and fine scale geographical and molecular assessment of stream fishes of the EZH freshwater ecoregion to assess levels of intraspecific genetic differentiation and explore if potential conflicts with their current taxonomic status can be identified.

## Materials and methods

### Ethics statement

This research was carried out following the evaluation and approval of the sampling protocols by the South African Institute for Aquatic Biodiversity (SAIAB) ethics committee (Ref: 2014/03). Permits to carry out this research were obtained from the Parks and Wildlife Authority of Zimbabwe.

### Study systems

The EZH freshwater ecoregion receives a mean annual precipitation of 900 to 3000 mm/a (Mazvimavi 2010) which sustain perennial flow in the rivers and streams of this region. There are four major river systems that drain this ecoregion: the Lower Zambezi, Pungwe, Save and Buzi systems (Fig. 1). Two tributaries of the Lower Zambezi (the Nyangombe and Kairezi rivers) drain the western and northern parts of this ecoregion. Three localities were sampled in the Nyangombe subcatchment and two localities were sampled in the Kairezi subcatchment (Fig. 1; Suppl. material 1). The Pungwe River flows eastwards from the EZH, and the river has a length of about 400 km from its source to its discharge point into the Indian Ocean near Beira in Mozambique. A total of 17 localities covering four major subcatchments of the Pungwe River system (i.e., the Rwera, Pungwe mainstream, Honde and Nyamukwarara) were sampled (Fig. 1; Suppl. material 1). Riparian zones of the Rwera and mainstream Pungwe subcatchments are covered with remnants of the Afromontane rainforests, whereas miombo woodlands are the dominant vegetation types in the riparian zones of the Honde and Nyamukwarara subcatchments. The Buzi River system has three eastward draining subcatchments (the Buzi, Rusitu and Revue). Two localities were sampled in the Rusitu subcatchment (Haruni and Upper Rusitu) which drains the Chimanimani Mountains. The Odzi River, a major subcatchment of the Save River system, drains southwards from the EZH (Fig. 1). Two localities were sampled in the Odzi subcatchment (the Nyamazi and Nyanyadzi rivers). A single locality in the Upper Save was also sampled to collect specimens and tissue samples from the type system of *Zaireichthys monomotapa* (Suppl. material 1).

### Fish sampling

Fishes were collected in December 2013 and 2014 using a Samus-725M electrofisher. Captured fishes were anaesthetised with clove oil, digitally photographed and a small piece of muscle tissue was dissected from the right side of each specimen and preserved in 95% ethanol in the field for genetic analysis. Tissue samples were stored at -80^o^C at the South African Institute for Aquatic Biodiversity (SAIAB), Grahamstown. Voucher specimens were fixed in 10% formalin in the field. They were then transferred through 10% and 50% to 70% ethanol for long-term storage. All voucher specimens were deposited into the fish collection facility at SAIAB as reference material. Species were identified using regional identification keys and their known geographic distributions according to Skelton (2001) and Marshall (2011).

### DNA Extraction, PCR and sequencing

DNA was extracted from preserved tissue using the salting out method (Sunnucks et al. 1996). DNA concentration was quantified using a Nanodrop ND-1000 (Nanodrop Technologies, Inc). A fragment of the mitochondrial cytochrome oxidase subunit I (COI) gene was amplified by polymerase chain reaction (PCR) using universal fish DNA barcoding primer sets: VF2-T1 and VR1-T1 (Ivanova et al. 2007), FishF1 and FishR1 or FishF2 and FishR2 (Ward et al. 2005). PCRs were performed with a Veriti 96 well thermal cycler (Applied Biosystems) and each reaction mixture (25 µL) contained 100–200 ng template DNA, 14.4 µL of water, 2.5 µL deoxynucleotide triphosphate (dNTP) (10 mM), 2.5 mM MgCl_2_, 2.5 µL PCR buffer (10X), 0.5 µL of each primer (20 pmol) and 0.1 µL *Taq* DNA polymerase (Southern Cross Biotechnology, Cape Town). The PCR amplification profile was 94 °C for 3 min, followed by 38 cycles of 94 °C for 30 s, 55 °C for 30 s and 72 °C for 50 s, and then final extension at 72 °C for 7 min. PCR products were purified with Exosap (Applied Biosystems), cycle-sequenced using BigDye Cycle Sequencing Kit (Applied Biosystems, Foster City, CA, USA) and sequenced at SAIAB using an ABI 3730xl DNA Analyzer (Applied Biosystems). Sequences generated from this study were submitted to GenBank (accession numbers: *Amphilius* (MH431952 - MH432017); *Chiloglanis* (MH432018 - MH432062); *Hippopotamyrus* (MH432063 - MH432086); *Zaireichthys* (MH432087 - MH432119); Suppl. material 1). BOLD sequences that were included in this study are also listed in Suppl. material 1.

### Data analyses

Sequences were cleaned, aligned and trimmed to equal lengths using the program SeqMan7.2.1 (DNASTAR, Madison, WI, USA). The appropriate models of sequence evolution for each genus were selected using the Akaike’s Information Criteria (AIC) (Burnham and Anderson 2002) as implemented in jModeltest 2 (Darriba et al. 2012). Phylogenetic relationships among unique haplotypes within each genus were inferred using MrBayes 3.1.2 (Ronquist et al. 2012). The analysis for each genus had two replicate searches of 10 million generations with four Markov chains. Trees were sampled every 1000 generations to obtain 10000 sampled trees. TRACER 1.5 (Rambaut and Drummond 2007) was used to assess if the chains had converged and determine the burn-in. We discarded 10% of the sampled trees as burn-in and the remainder were used to calculate the consensus tree and Bayesian posterior probabilities. Model corrected genetic distances between unique lineages identified for each genus were calculated using PAUP (Swofford 2003). To explore the possible taxonomic distinctiveness of the genetic lineages that will be uncovered from the Eastern Highlands, sequences of topotypes (i.e., samples collected from or within the vicinity of the type localities of currently described species), whenever available, were also included in the analyses.

The General Mixed Yule Coalescent (GMYC) method (Pons et al. 2006) was used to delineate candidate species or operational taxonomic units (OTUs). The GMYC is a robust method that models branching thresholds for intraspecific (coalescent) and interspecific (speciation/diversification) patterns (Fujisawa and Barraclough 2013). This approach has been widely applied in a number of studies to identify cryptic diversity within morphologically defined species (e.g., Crivellaro et al. 2017; Grabowski et al. 2017; Kordbacheh et al. 2017). The ultrametric trees for GMYC analyses were estimated in BEAST (Drummond and Rambaut 2007) using two priors (Yule model and Coalescent model with constant population size) and two rates of molecular evolution (constant and relaxed clock). For each of the four genera considered in the present study, we compared three trees which were built based on the following combinations of priors and rates of molecular evolution: (i) yule model and a constant clock, (ii) yule model and a relaxed clock, and (iii) coalescent model with constant population size and a constant clock. The GMYC analyses were conducted with the package ‘splits’ (Species Limits by Threshold Statistics (http://r-forge.r-project.org/projects/splits) using R v.3.4.1 (R Development Core Team 2011).

## Results

A total of 153 COI sequences were generated from individuals representing five currently recognised species considered in the present study from the Eastern Zimbabwe Highlands freshwater ecoregion: *Chiloglanis neumanni* (43 sequences), *Amphilius natalensis* (37 sequences), *A. uranoscopus* (26 sequences), *Zaireichthys monomotapa* (32 sequences) and *Hippopotamyrus ansorgii* (16 sequences). An additional 107 sequences were downloaded from BOLD for comparison (Suppl. material 1). Read lengths of all sequences used in this study were over 530 bp long. No stop codons were observed when examining amino acid translations, indicating that the amplified domains were functional mitochondrial copies.

### Genetic structure in *Chiloglanis neumanni*

The COI dataset for *Chiloglanis* consisted of 73 sequences (included 30 BOLD sequences; Suppl. material 1) and an edited alignment of 534 bp with 175 polymorphic sites which resulted in 48 unique haplotypes. Bayesian analysis divided haplotypes of *C. neumanni* from the EZH ecoregion into two well-supported clades (Fig. 2a). Estimates of the number of candidate species using GMYC ranged from eight to 16, excluding outgroups (Fig. 2a). The coalescent model with constant population size and a constant clock (CONC) gave the most conservative estimate of the number of candidate species, and these were largely consistent with the clades inferred using the Bayesian analysis, with the exception of one sample in the *C.* sp. ‘rough skin’ clade and two samples in the *C.* sp. ‘dwarf’ clade which were assigned to separate clusters from these two major clades (Fig. 2a). Both the Yule model and a constant clock (YULE), and the Yule model and a relaxed clock (RELA) clearly overestimated the number of candidate species due to over splitting of samples in the *C.* sp. ‘rough skin’ and *C.* sp. ‘dwarf’ clades (Fig. 2a). A conservative approach was therefore considered in the present study, and six candidate species or molecular operation taxonomic units (MOTUs) were delimited within *C. neumanni* from the EZH ecoregion and the adjacent Shire and Ruo rivers in Malawi (Fig. 2a). The first major clade in Fig. 2a contained four candidate species, *C.* sp. ‘rough skin’, *C.* sp. ‘Zambezi’, *C.* sp. ‘Pungwe’, and *C.* sp. ‘Shire’, with model corrected genetic divergences between these candidate species ranging from 1.35–7.60% (Table 1). The second major clade contained two candidate species, *C.* sp. ‘dwarf’ and *C.* sp. ‘Nyangombe’ which were separated by 4.01–5.27% sequence divergence.

**Figure 2a.**
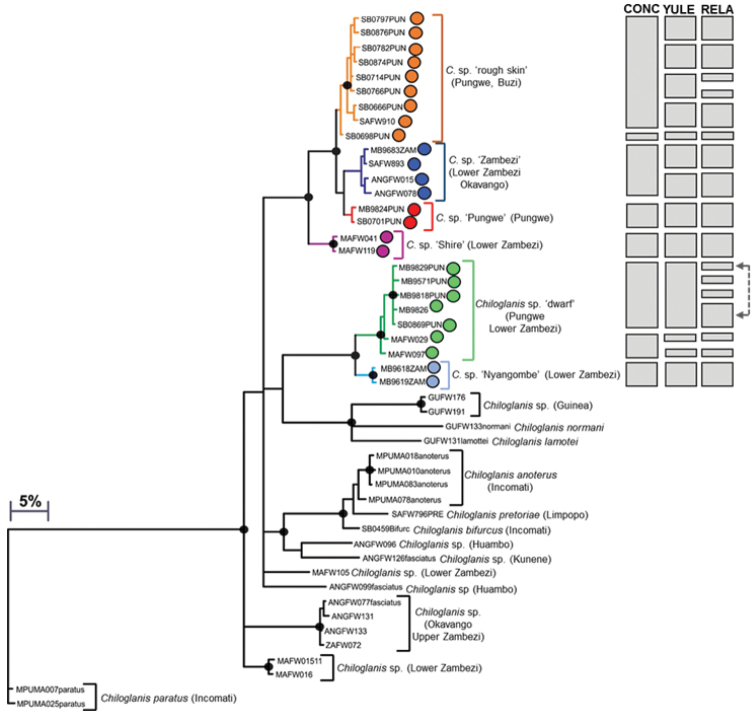
Bayesian phylogenetic tree based on mtDNA cytochrome oxidase sub unit I (COI) sequences showing the candidate species or molecular operational taxonomic units (MOTUs) identified within *Chiloglanis neumanni* from the Eastern Zimbabwe Highlands freshwater ecoregion. Well supported nodes are shown by a solid circle. The indicated candidate species or MOTUs were identified using the GMYC method based on trees that were built using three different combinations of priors and rates of molecular evolution: (i) coalescent model with constant population size and a constant clock (CONC), (ii) yule model and a constant clock (YULE) and (iii) yule model and a relaxed clock (RELA).

**Table 1.** Mitochondrial COI genetic distances (%) between *Chiloglanis* lineages from the Eastern Highlands of Zimbabwe and selected species from southern Africa.

	*C.* sp. ‘rough skin’	*C.* ‘Zambezi’	*C.* sp. ‘Pungwe’	*C.* sp. ‘Shire’	*C.* sp. ‘dwarf’	*C.* sp. ‘Nyangombe’	*C. pretoriae*	*C. anoterus*
*C.* sp. ‘rough skin’	0.00–1.38							
*C.* sp. ‘Zambezi’	2.36–4.24	0.00–0.76						
*C.* sp. ‘Pungwe’	1.35–2.67	2.19–3.12	0.18					
*C.* sp. ‘Shire’	5.01–7.14	6.33–7.60	6.47–7.04	0.19				
*C.* sp. ‘dwarf’	16.88–19.86	17.13–19.08	18.30–20.10	18.48–20.14	0.00–1.33			
*C.* sp. ‘Nyangombe’	13.95–16.04	13.25–14.54	15.37–16.15	16.86–17.64	4.01–5.27	0.18		
*C. pretoriae*	19.24–20.89	18.94–20.18	19.24–19.76	18.38–18.81	16.59–17.26	17.45–17.83		
*C. anoterus*	15.77–19.70	16.44–19.03	16.62–19.63	17.04–20.04	18.60–22.03	17.55–19.88	4.29–5.12	0.00–1.98
*C. bifurcus*	17.40–18.90	16.59–17.34	18.34–18.83	15.79–16.18	16.16–17.01	17.20–17.57	5.14	2.85–3.59

*Chiloglanis* sp. ‘rough skin’ comprised haplotypes from the Pungwe and Buzi river systems (Fig. 2b). *Chiloglanis* sp. ‘Zambezi’ contained haplotypes from the Nyangombe River in the EZH ecoregion, as well from a tributary that flows into Lake Cahora Bassa and from the Cubango River which is part of the Okavango River system (Fig. 2b). *Chiloglanis* sp. ‘Pungwe’ contained haplotypes that were confined to the Pungwe River system (Fig. 2c). *Chiloglanis* sp. ‘Shire’ was confined to the Shire and Ruo rivers (Fig. 2b). *Chiloglanis* sp. ‘dwarf’ was distributed in the Pungwe and Ruo rivers, while *C.* sp. ‘Nyangombe’ was only recorded from the Nyangombe subcatchment during the present study (Fig. 2c). The genetic divergences between almost all the candidate species identified from the present study are consistent with interspecific genetic divergences between morphologically distinguishable *Chiloglanis* species, for example *C. anoterus* and *C. bifurcus* from the Inkomati River system which are separated by 2.85–3.59% COI sequence divergence (Table 1). It is also important to note that all the candidate species identified from the EZH freshwater ecoregion are deeply divergent from *C. pretoriae* (16.59–20.89% sequence divergence; Table 1; Fig. 2a), a name that was previously assigned to the EZH ecoregion suckermouth catlets. Unfortunately, sequences of both *C. neumanni* sensu stricto and *C. emarginatus*, two other species names that were previously used for the EZH freshwater ecoregion suckermouth catlets, were not available for comparison in the present study.

**Figure 2b.**
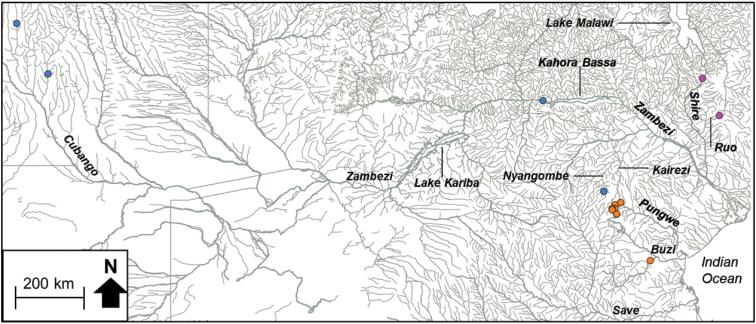
The distribution of *Chiloglanis* sp. ‘rough skin’ (orange circle), *Chiloglanis* sp. ‘Zambezi’ (blue circle) and *Chloglanis* sp. ‘Shire’ (purple circle) in the Eastern Zimbabwe Highlands freshwater ecoregion and adjacent areas.

**Figure 2c.**
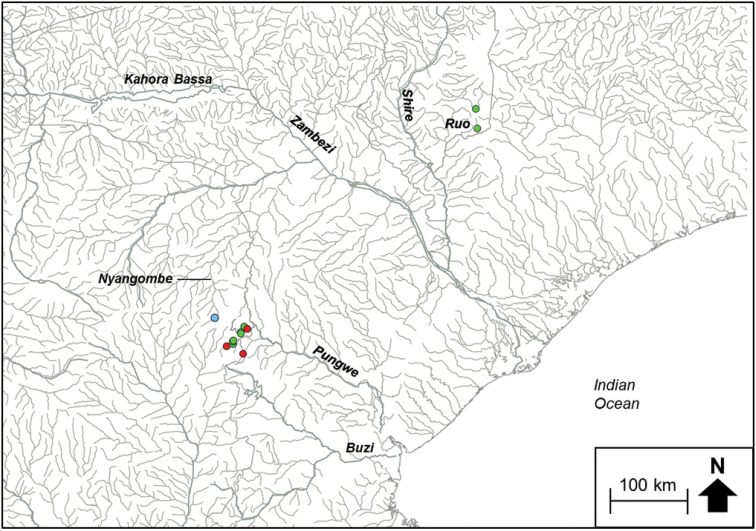
The distribution of *Chiloglanis* sp. ‘dwarf’ (green circle), *Chiloglanis* sp. ‘Nyangombe’ (blue circle) and *Chloglanis* sp. ‘Pungwe’ (red circle) in the Eastern Zimbabwe Highlands freshwater ecoregion and adjacent areas.

### Genetic structure in *Amphilius*

The COI dataset for *Amphilius* consisted of 79 sequences (included 16 BOLD sequences; Suppl. material 1) and an edited alignment of 572 bp with 166 polymorphic sites which resulted in 34 unique haplotypes. Bayesian analysis divided samples of *A. natalensis* and *A. uranoscopus* into two separate and well-supported clades (Fig. 3a). Estimates of the number of candidate species within *A. natalensis* using GMYC ranged from one to six (Fig. 3a). Yule model and a relaxed clock (RELA) clearly underestimated the number of candidate species in *A. natalensis* sensu lato due to the geographic isolation and disjunct distribution of the populations within this ‘species’. Five candidate species were therefore delineated based on trees generated using the Yule model and a constant clock (YULE), which gave the next most conservative estimate on the number of species (Fig. 3a). Two of these candidate species, *A.* sp. ‘*natalensis* Pungwe’ and *A.* sp. ‘*natalensis* Buzi’ are confined to the Pungwe and Buzi river systems, respectively (Fig. 3b). These two candidate species are allopatrically distributed and are separated by substantial genetic divergence (Fig. 3a; 6.86–8.58% genetic divergence; Table 2). Divergences within candidate species ranged from 0.00–0.72% in *A.* sp. ‘Pungwe’ and from 0.00–1.30% in *A.* sp. ‘Buzi’. *Amphilius* sp. ‘Pungwe’ was collected from multiple localities in the Pungwe River system and a single locality in the Kairezi River, a tributary of the Lower Zambezi, while *A.* sp. ‘Buzi’ was collected from the Haruni, Rusitu, and the mainstream Buzi River (Fig. 3b). The present study revealed that *A.* sp. ‘Pungwe’ and *A.* sp. ‘Buzi’ are genetically distinct (10.44–12.71% sequence divergence, Table 2; Fig. 3a) from *A. natalensis* sensu stricto based on a specimen collected from the Umgeni River (the type system for *A. natalensis*) in South Africa.

**Figure 3a.**
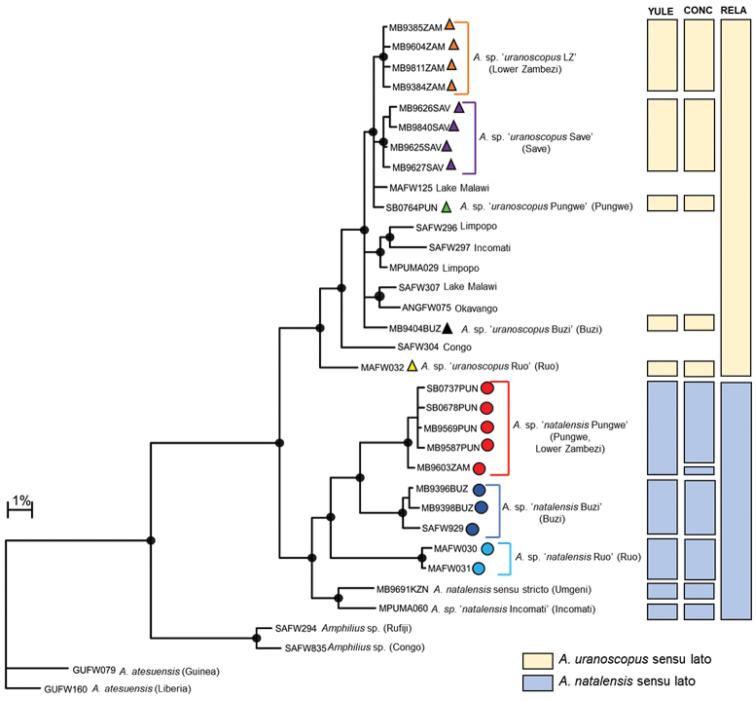
Bayesian phylogenetic tree based on mtDNA cytochrome oxidase sub unit I (COI) sequences showing the candidate species or molecular operational taxonomic units (MOTUs) identified within *Amphilius uranoscopus* and *A. natalensis* from the Eastern Highlands of Zimbabwe. Well supported nodes are shown by a solid circle. The indicated candidate species or OTUs were identified using the GMYC method based on trees that were built using three different combinations of priors and rates of molecular evolution: (i) yule model and a constant clock (YULE), (ii) coalescent model with constant population size and a constant clock (CONC) and (iii) yule model and a relaxed clock (RELA).

**Table 2.** Mitochondrial COI genetic distances (%) between lineages of *Amphilius natalensis* sensu lato identified from the Eastern Highlands of Zimbabwe and other lineages within this ‘species’ from selected populations in southern Africa.

	*A.* sp. ‘Pungwe’	*A.* sp. ‘Buzi’	*A.* sp. ‘Ruo’	*A. natalensis* s.s
*A.* sp. ‘Pungwe’	0.00–0.72			
*A.* sp. ‘Buzi’	6.86–8.58	0.00–1.30		
*A.* sp. ‘Ruo’	15.20–15.63	14.32–16.77	0.35	
*A. natalensis* s.s	11.16–12.71	10.44–11.06	13.05–13.91	-
*A.* sp. ‘Inkomati’	13.16–14.05	11.65–12.32	14.42–15.33	4.44

**Figure 3b.**
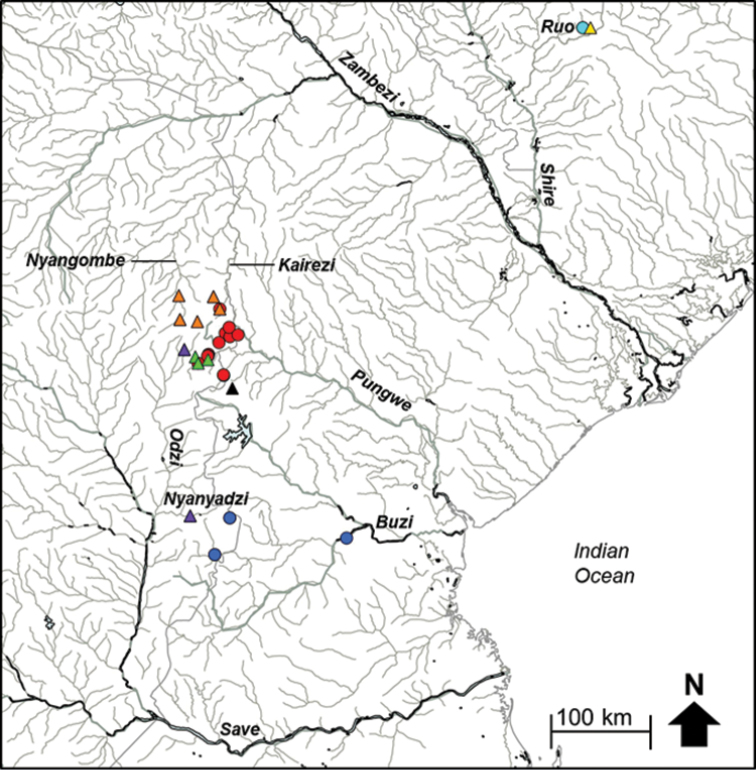
The distribution of *Amphilius* sp. ‘natalensis Buzi’ (dark blue circle), *Amphilius* sp. ‘natalensis Pungwe’ (red circle) and *Amphilius* sp. ‘natalensis Ruo’ (light blue circle), *Amphilius* sp. ‘uranoscopus Save’ (purple triangle), *Amphilius* sp. ‘uranoscopus Buzi’ (black triangle), *Amphilius* sp. ‘uranoscopus Pungwe’ (green triangle), *Amphilius* sp. ‘uranoscopus Zambezi’ (orange triangle) and *Amphilius* sp. ‘uranoscopus Ruo’ (yellow triangle) in the Eastern Zimbabwe Highlands freshwater ecoregion and adjacent areas.

Bayesian analysis revealed a well-supported shallow clade corresponding to the species currently recognised as *A. uranoscopus* in southern Africa (Fig. 3a). Estimates of the number of candidate species within *A. uranoscopus* using GMYC gave consistent results for the EZH ecoregion (four candidate species) based on both the Yule model and a constant clock (YULE) and the coalescent model with constant population size and a constant clock (CONC) models (Fig. 3a). All the candidate species within *A. uranoscopus* from the EZH ecoregion are allopatrically distributed. Haplotypes within *Amphilius* sp. ‘*uranoscopus* Save’ were collected from the Nyamazi River, a tributary of the Odzi River, and from the mainstream Nyanyadzi River (Fig. 3b). Both *A.* sp. ‘*uranoscopus* Buzi’ and *A.* sp. ‘*uranoscopus* Pungwe’ were confined to the Buzi and Pungwe river systems, respectively (Fig. 3b). Haplotypes within *A.* sp. ‘*uranoscopus* LZ’ occurred in the Nyangombe and Kairezi rivers, which are both tributaries of the Lower Zambezi (Fig. 3b). The candidate species within *A. uranoscopus* sensu lato had shallow divergences among them (0.54–2.56% sequence divergence; Table 3) compared to the deep divergences found among lineages within *A. natalensis* sensu lato (Table 2). Notable exceptions were samples of *A. uranoscopus* collected from the Ruo River (BOLD sequence MAFW032) which is the type river for *A. hargeri* and from the Cubango River (BOLD sequence ANGFW075) which is the type river for *A. cubangoensis* which were respectively deeply and moderately divergent from all the other lineages within the *A. uranoscopus* sensu lato clade (Table 3). The analysis also showed that the Ruo and Cubango samples of *A. uranoscopus* were genetically distinct, being separated by 7.23% (Table 3).

**Table 3.** Mitochondrial COI genetic distances (%) between populations of *Amphilius uranoscopus* from the Eastern Highlands of Zimbabwe and selected localities in southern Africa.

	*A.* sp. ‘Zambezi’	*A.* sp. ‘Pungwe’	*A.* sp. ‘Buzi’	*A.* sp. ‘Save’	*A.* sp. ‘Ruo’
*A.* sp. ‘Zambezi’	0.00–0.36				
*A.* sp. ‘Pungwe’	0.72–0.92	-			
*A.* sp. ‘Buzi’	1.88–2.13	2.06	-		
*A.* sp. ‘Save’	0.54–1.34	0.73–1.13	2.07–2.56	0.00–0.54	
*A.* sp. ‘Ruo’	5.21–5.86	6.07	6.21	6.12–6.81	-
*A.* sp. ‘Cubango’	2.42–2.89	2.64	2.71	2.67–3.17	7.23

### Genetic structure in *Zaireichthys monomotapa*

The edited alignment of 47 *Zaireichthys* sequences (included 15 BOLD sequences, Suppl. material 1) was 534 base pairs in length with 151 polymorphic sites which resulted in 21 unique haplotypes. GMYC analyses delimited *Z. monomotapa* sensu stricto which is distributed in the Save and Pungwe river systems, and identified one candidate species from the EZH ecoregion, *Z.* sp. ‘slender’, which occurred in the mainstream Nyangombe River and its tributary, the Chidiya River (Fig. 4a, 4b). BOLD sequences (SAFW899, SAFW900, SAFW905, SAFW907, SAFW908) for individuals collected from three streams which flow into Lake Cahora Bassa, as well as sequences MAFW12911 and MAFW13611 for individuals collected from two tributaries which flow into Lake Malawi also belonged to this clade (Fig. 4b; Suppl. material 1), indicating that this taxon may be widespread in the Lower Zambezi and Lake Malawi catchments (Fig. 4b). *Zaireichthys monomotapa* sensu stricto and *Z.* sp. ‘slender’ were separated by 9.26–11.00% sequence divergence, while divergences within these taxa ranged from 0.0–0.96%. *Zaireichthys* sp. ‘leopard spot’, a candidate species from the Ruo River, was basal to *Z. monomotapa* sensu stricto (Fig. 4a), with a divergence of 3.22–3.80% between these two sister taxa. A second candidate species, *Z.* sp. ‘Chilwa’, within the *Z. monomotapa* complex occurred in the Montepuez River system in northern Mozambique and the Lake Chilwa system in Malawi is also shown in Fig. 4a.

**Figure 4a.**
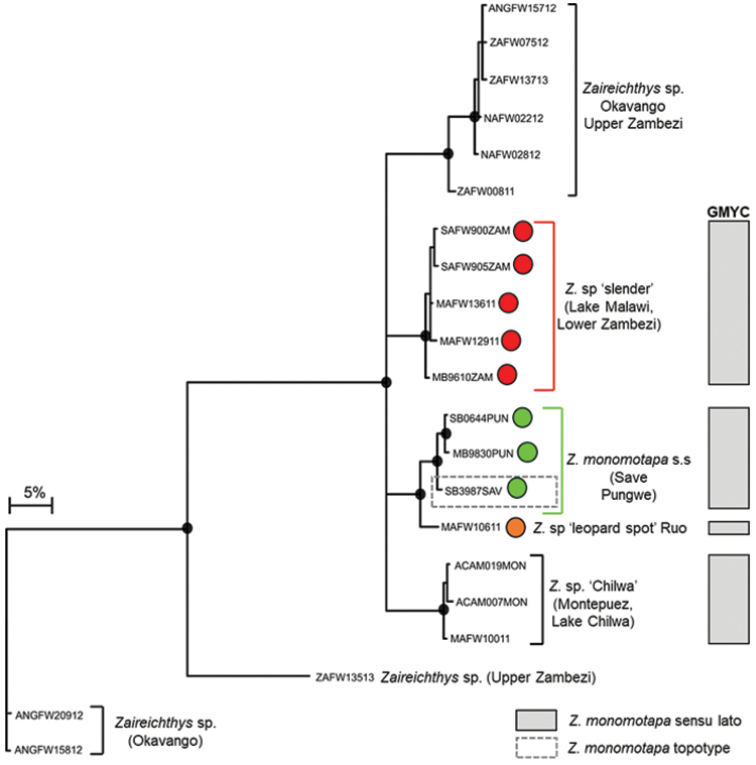
Bayesian phylogenetic tree based on mtDNA cytochrome oxidase sub unit I (COI) sequences showing the candidate species or molecular operational taxonomic units (MOTUs) identified within *Zaireichthys monomotapa* from the Eastern Zimbabwe Highlands freshwater ecoregion. Well supported nodes are shown by a solid circle. The indicated candidate species or MOTUs were identified using the GMYC method based on trees that were built using three different combinations of priors and rates of molecular evolution: (i) yule model and a constant clock, (ii) yule model and a relaxed clock, and (iii) coalescent model with constant population size and a constant clock.

**Figure 4b.**
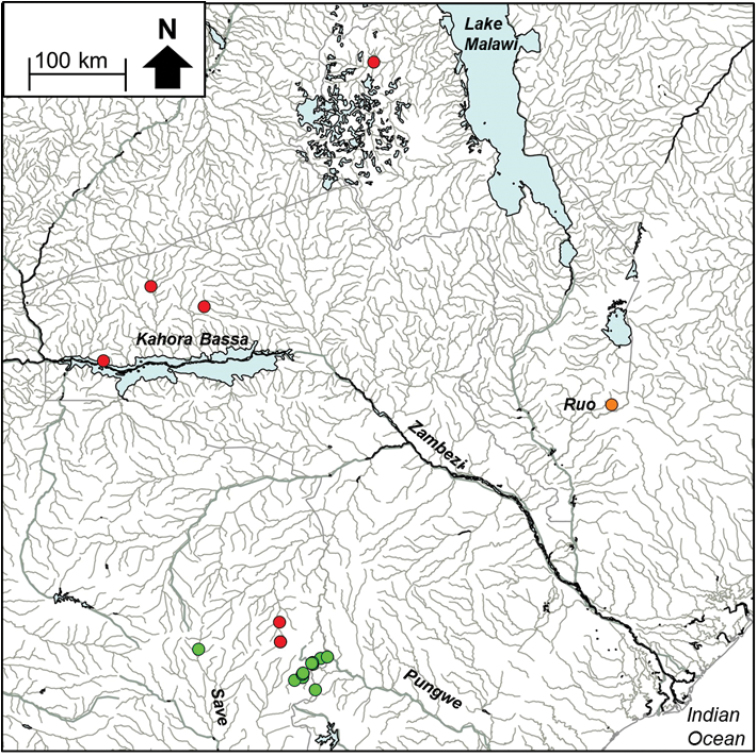
The distribution of *Zaireichthys monomotapa* sensu stricto (green circle), *Zaireichthys* sp. ‘slender’ (red circle) and *Zaireichthys* sp. ‘leopard spot’ (orange circle) in the Eastern Zimbabwe Highlands freshwater ecoregion and adjacent areas.

### Genetic structure in *Hippopotamyrus ansorgii*

The edited alignment of 27 *Hippopotamyrus* sequences (included 11 BOLD sequences, Suppl. material 1), including a single *Cyphomyrus cubangoensis* sequence, was 580 base pairs and had 88 polymorphic sites which defined 17 haplotypes. Phylogenetic inference assigned the Pungwe and Buzi populations of *H. ansorgii* to two distinct clades that were separated by 2.85–3.27% divergence (Fig. 5a; Table 4). Divergences within lineages were 0.0–0.35% for *H.* sp. ‘Pungwe’ and 0.00–1.25% for *H.* sp. ‘Buzi’. GMYC analysis identified three clusters (or candidate species): (i) samples from the Kwanza, Upper Zambezi and the Buzi River system, (ii) samples from the Kunene River system and the Ruo River, and (iii) samples from the Pungwe River system represented a candidate species, *H.* sp. ‘Pungwe’ (Fig. 5a, 5b). The genetic divergences between lineages within these clusters are presented in Table 4.

**Figure 5a.**
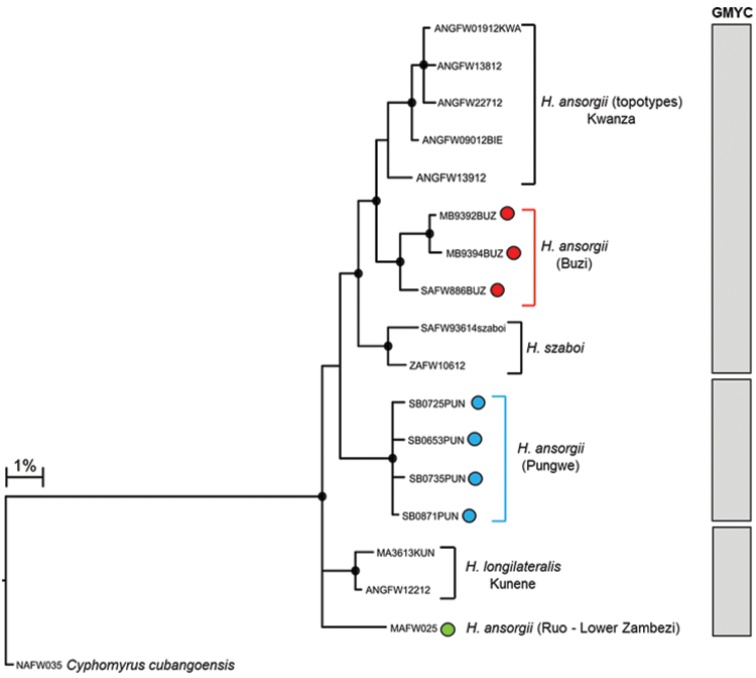
Bayesian phylogenetic tree based on mtDNA cytochrome oxidase sub unit I (COI) sequences showing the candidate species or molecular operational taxonomic units (MOTUs) identified within *Hippopotamyrus ansorgii* from the Eastern Zimbabwe Highlands freshwater ecoregion. Well supported nodes are shown by a solid circle. The indicated candidate species or MOTUs were identified using the GMYC method based on trees that were built using three different combinations of priors and rates of molecular evolution: (i) yule model and a constant clock, (ii) yule model and a relaxed clock, and (iii) coalescent model with constant population size and a constant clock.

**Table 4.** Mitochondrial COI genetic distances (%) between lineages and species of in the *Hippopotamyrus ansorgii* complex from southern Africa.

	*H.* sp. ‘Pungwe’	*H.* sp. ‘Buzi’	*H.* sp. ‘Ruo’	*H.* sp. ‘Kwanza’	*H. szaboi*
*H.* sp. ‘Pungwe’	0.00–0.35				
*H.* sp. ‘Buzi’	2.85–3.27	0.00–1.25			
*H.* sp. ‘Ruo’	3.50–3.71	4.17–4.40	-		
*H.* sp. ‘Kwanza’	3.50–3.96	1.64–2.63	3.50–3.96	0.17–1.73	
*H. szaboi*	3.03–3.25	2.42–2.63	3.92	2.61–3.03	-
*H. longilateralis*	2.64–3.47	3.06–3.74	2.85–3.27	2.44–3.74	3.23–3.68

**Figure 5b.**
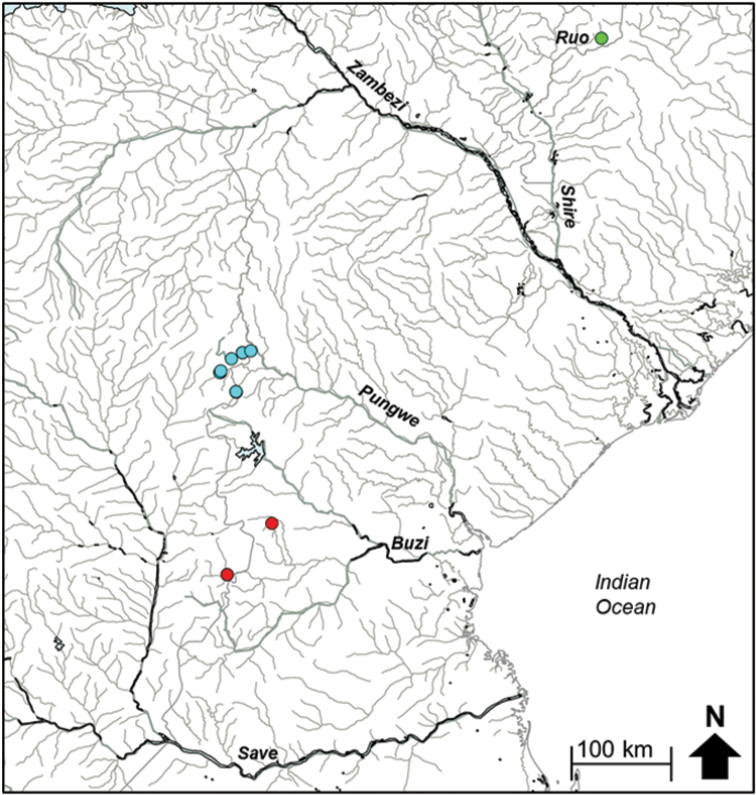
The distribution of *Hippopotamyrus* sp. ‘Buzi’, (red circle), Hippopotamyrus sp. ‘Pungwe’ (blue circle) and *Hippopotamyrus* sp. ‘Ruo’ (green circle) in the Eastern Zimbabwe Highlands freshwater ecoregion and adjacent areas.

## Discussion

### Hidden diversity in stream fishes of the Eastern Zimbabwe Highlands freshwater ecoregion

This study represents the first fine scale geographical survey and genetic exploration to determine the extent of hidden diversity in stream fishes of the Eastern Zimbabwe Highlands (EZH) freshwater ecoregion. Species delimitation assessment using the GMYC method revealed existence of 16 molecular operational taxonomic units (MOTUs) or putative species in five currently recognised nominal species (i.e., six MOTUs in *Chiloglanis neumanni*, two MOTUs in *Amphilius natalensis*, four MOTUs in *A. uranoscopus*, two MOTUs in *Z. monomotapa* and two MOTUs in *H. ansorgii*) collected from the EZH freshwater ecoregion. Given that all these five ‘species’ (with the exception of *Z. monomotapa* sensu stricto) were described from systems outside the EZH freshwater ecoregion, 15 of the 16 identified MOTUs within these ‘species’ are likely to represent new species that were previously unrecognised by science. Although the GMYC is a robust method for species delimitation (Fujisawa and Barraclough 2013), the entities identified in the present study were not designated as ‘distinct species’, but rather considered to represent ‘candidate or potential species’ pending critical evaluation using integrative approaches that combine molecular, morphological and ecological data to determine their taxonomic integrity. Ongoing assessments by the authors have revealed consistent morphological differences between *Chiloglanis* sp. ‘rough skin’ and *C.* sp. ‘dwarf’, and formal descriptions of these newly identified species are underway.

While the present study’s main focus was on the EZH freshwater ecoregion, it is important to indicate that the diversity of stream fishes in the broader Eastern Afromontane Region may be much higher than currently documented as highlighted by the presence of other candidate species outside the EZH freshwater ecoregion. These include *Amphilius* sp. ‘Ruo’, *Zaireichthys* sp. ‘Chilwa’ and *Hippopotamyrus* sp. ‘Ruo’, as well as lineages within the *A. uranoscopus* complex. Additional fine-scale geographic surveys are therefore required to fill missing sampling gaps to more accurately map the distribution ranges of these lineages and potentially identify additional hidden diversity. Results of the present study also showed that samples collected from the type localities of the three synonyms of *A. uranoscopus* from southern Africa, *A. hargeri* (BOLD sequence MAFW032), *A. brevidorsalis* (sequence MB9404BUZ) and *A. cubangoensis* (BOLD sequence ANGFW075) were genetically differentiated from other populations of *A. uranoscopus* sensu lato (see Fig. 4a). This warrants further taxonomic investigations to determine whether these synonyms represent valid species that may need to be resurrected.

The existence of such high taxonomic diversity within a small portion of the Eastern Afromontane Region is consistent with findings from a number of previous studies that have uncovered substantial hidden diversity and narrow range endemic species (or lineages) within several stream fishes that were previously thought to have wide geographic ranges in southern Africa (e.g., Chakona et al. 2013; Goodier et al. 2011; Maake et al. 2014; Morris et al. 2015; Swartz et al. 2007, 2009; Wishart et al. 2006). Results of the present study thus add to the growing board of evidence that shows that a large proportion of freshwater fishes in southern Africa remain scientifically undocumented, because many river systems remain poorly explored as much of the previous research effort and application of molecular approaches has predominantly focussed on fishes of the Cape Fold Ecoregion (Ellender et al. 2017). Despite being one of the geographically and taxonomically well explored regions in southern Africa, new species and deeply divergent genetic lineages continue to be discovered within almost all fish taxonomic groups of the Cape Fold Ecoregion (Chakona and Swartz, 2013; Chakona and Skelton, 2017; Chakona et al. 2013, 2014; Wishart et al. 2006), and estimates indicate that there are about 43 undescribed species within the 21 currently recognised fish species of this region (Linder et al. 2010). The discovery of hidden diversity in the EZH freshwater ecoregion adds to the growing evidence for the existence of high species-level diversity within a number of fish species from high altitude streams in southern, east and west Africa (e.g., Friel and Vigliotta 2011; Morris et al. 2016; Schmidt and Pezold 2011; Schmidt et al. 2014, 2015, 2016; Thomson 2013; Thomson and Page 2010). Given that many regions in southern Africa, in particular the subtropical and tropical river systems, have not been adequately explored, and the use of modern approaches for rapid species discovery remains very limited, additional diversity is likely to remain hidden within other wide-ranging ‘species’ in the region.

### Conservation implications

Findings from the present study have considerable implications for aquatic biodiversity conservation in the EZH freshwater ecoregion, and the broader Eastern Afromontane region. Existence of such high cryptic diversity within five ‘species’ from a few mountain tributaries which represent a very small portion of the Eastern Afromontane region indicates that the overall conservation value of this globally important biodiversity hotspot has been severely underestimated. This is because the current biodiversity estimates and level of endemism in this region does not include cryptic diversity within stream fishes. Because many of the stream fishes from the EZH freshwater ecoregion are thought to have wide geographic ranges, they are considered to be of least conservation concern (Tweddle et al. 2009). This has resulted in stream fishes being neglected from ongoing conservation efforts in the EZH freshwater ecoregion, and the broader Eastern Afromontane region. This can be seen in the calls for research funding, where the primary focus is on other vertebrate groups such as birds, amphibians, reptiles and small mammals (e.g., see http://www.cepf.net/grants/project_database/Pages/default.aspx#). The present study uncovers a classic example where underestimation of taxonomic diversity and poor understanding of the spatial distribution of species has misdirected conservation efforts, to the extent that the EZH freshwater ecoregion is currently not listed among the priority freshwater Key Biodiversity Areas within the Eastern Afromontane Biodiversity Hotspot (see CEPF 2012). This is however unfortunate because the highly sensitive Afromontane streams and rivers in this region have been severely transformed and are experiencing ongoing human impacts, including illegal mining activities, deforestation, increased sedimentation, uncontrolled burning and introduction of non-native invasive piscivorous species (Kadye and Magadza 2008; Kadye et al. 2013). For example, the rocky streams which drain the Chimanimani Mountains used to have intact indigenous riparian vegetation, had clear water and perennial flow, but surveys in 2013 revealed that human encroachment, increased agricultural activities and the associated loss of riparian vegetation has transformed these streams into sluggish flowing, and highly turbid and heavily silted habitats. This increases the risk of losing sensitive fish species and other aquatic taxa from the EZH freshwater ecoregion whose value as an endemic hotspot has been previously underestimated.

## Supplementary materials

Supplementary material 1 Evidence of hidden diversity and taxonomic conflicts in five stream fishes from the Eastern Zimbabwe Highlands freshwater ecoregion Data type: (Mitochondrial COI sequences, sample size, occurrence) Explanation note: Locality information, sample size, BOLD and GenBank accession numbers for mitochondrial COI sequences, and the candidate species identified in *Amphilius natalensis*, *A. uranoscopus*, *Chiloglanis neumanni*, *Hippopotamyrus ansorgii* and *Zaireichthys monomotapa* from the Eastern Zimbabwe Highlands freshwater ecoregion. This is an open access article distributed under the terms of the Creative Commons Attribution License (CC BY 4.0), which permits unrestricted use, distribution, and reproduction in any medium, provided the original author and source are credited.Albert Chakona, Wilbert T. Kadye, Taurai Bere, Daniel N. Mazungula, Emmanuel Vreven
